# Supplementary material for: KMT2A associates with PHF5A-PHF14-HMG20A-RAI1 subcomplex in pancreatic cancer stem cells and epigenetically regulates their characteristics
Source: Nat Commun. 2023 Sep 14;14:5685. doi: 10.1038/s41467-023-41297-4 (PMC10502114; doi:10.1038/s41467-023-41297-4)
Supplement: Supplementary file 1 — Supplementary Information [file 41467_2023_41297_MOESM1_ESM.pdf]

Supplementary Information File

KMT2A associates with PHF5A-PHF14-HMG20A-RAI1 subcomplex in pancreatic cancer stem cells and epigenetically regulates their characteristics

Mai Abdel Mouti[1], Siwei Deng[1], Martin Pook[1,2], Jessica Malzahn[1], Aniko Rendek[3], Stefania Militi[1], Reshma Nibhani[1], Zahir Soonawalla[4], Udo Oppermann[1], Chang-il Hwang[5], Siim Pauklin[1,*]

[1]Botnar Research Centre, Nuffield Department of Orthopaedics, Rheumatology, and Musculoskeletal Sciences, University of Oxford, UK
[2]Institute of Biomedicine and Translational Medicine, Faculty of Medicine, University of Tartu, Estonia
[3]Department of Histopathology, Oxford University Hospitals NHS Foundation Trust, Oxford, UK
[4]Department of Hepatobiliary and Pancreatic Surgery, Oxford University Hospitals NHS, Oxford, UK
[5]Department of Microbiology and Molecular Genetics, University of California Davis, USA

*Correspondence: siim.pauklin@ndorms.ox.ac.uk

Supplementary Figure S1

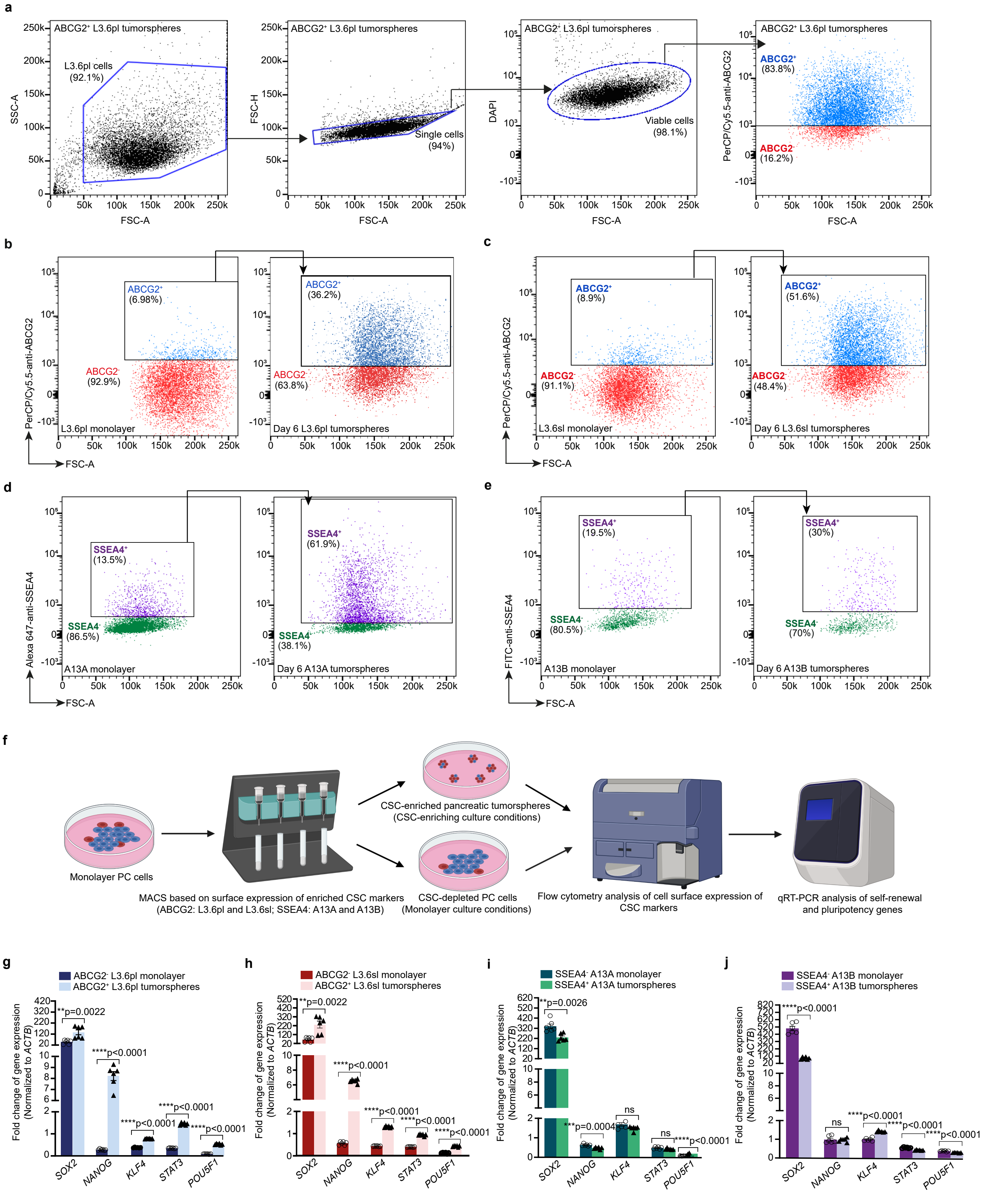

Supplementary Figure S1. Identification and characterization of PCSCs

a, The gating strategy for flow cytometry analysis of ABCG2 enrichment in ABCG2^{+} L3.6pl tumorspheres. PerCP-Cy5.5 Mouse IgG2b, κ isotype control was used as a negative control to identify and gate the positive cell population.

b-e, Flow cytometry dot plots illustrating enriched CSC surface markers in day 6 L3.6pl (**b, right panel**), L3.6sl (**c, right panel**), A13A (**d, right panel**), and A13B (**e, right panel**) tumorspheres as compared to L3.6pl (**b, left panel**), L3.6sl (**c, left panel**), A13A (**d, left panel**), and A13B (**e, left panel**) monolayer cells. Both isotype and fluorescence minus one (FMO) controls were used to gate the positive cell population.

f, Schematic diagram illustrating the workflow for qRT-PCR analysis of self-renewal and pluripotency genes in CSC-enriched pancreatic tumorspheres versus CSC-depleted monolayer PC cells sorted by MACs. The schematic illustration was created with Biorender scientific illustration software.

g-j, qRT-PCR analysis of self-renewal and pluripotency genes in ABCG2^{+} L3.6pl (**g**), ABCG2^{+} L3.6sl (**h**), SSEA4^{+} A13A (**i**), and SSEA4^{+} A13B (**j**) tumorspheres versus their respective CSC marker-depleted monolayer cells. *ACTB* was used for the normalization of mRNA expression levels. Data are representative of 3 biologically independent experiments (6 technical replicates per biological replicate) and presented as the mean value ± SEM. P values were calculated using a two-tailed *t*-test with Welch's correction for unequal variances.

Supplementary Figure S2

a

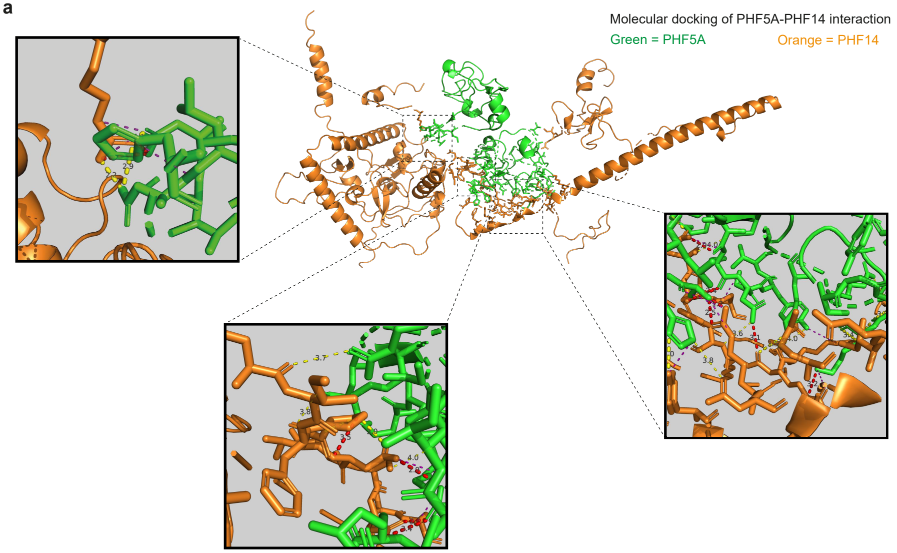

b

PHF5A-PHF14 interface pairs	Distance (Angstroms)	PHF5A-PHF14 interface pairs	Distance (Angstroms)
2A - 610A	2.974	36A - 602A	3.618
2A - 626A	1.678	36A - 605A	3.356
3A - 588A	4.42	36A - 607A	2.26
3A - 593A	2.816	37A - 598A	4.335
4A - 593A	3.81	37A - 601A	4.091
4A - 613A	3.676	37A - 602A	3.4
4A - 619A	2.692	42A - 761A	3.551
4A - 623A	2.772	53A - 637A	3.084
5A - 623A	2.34	53A - 641A	3.691
5A - 626A	4.596	54A - 634A	3.67
5A - 627A	3.839	54A - 637A	3.063
6A - 588A	4.909	57A - 633A	4.623
8A - 626A	4.775	57A - 636A	4.255
9A - 592A	3.873	57A - 637A	3.005
10A - 592A	4.275	57A - 640A	3.57
10A - 594A	3.332	61A - 630A	4.409
12A - 591A	3.704	61A - 633A	2.549
12A - 592A	4.02	62A - 633A	3.467
12A - 593A	4.976	62A - 637A	3.025
12A - 594A	2.857	63A - 633A	3.696
17A - 760A	4.733	63A - 637A	4.986
19A - 760A	4.104	68A - 761A	2.648
24A - 607A	3.289	70A - 761A	3.408
24A - 884A	4.802	72A - 598A	4.425
24A - 886A	2.3	74A - 597A	3.88
25A - 607A	3.367	74A - 598A	3.479
25A - 608A	2.642	75A - 596A	3.224
27A - 607A	4.777	75A - 598A	3.665
27A - 886A	3.31	78A - 595A	4.981
29A - 607A	2.742	78A - 597A	3.802
29A - 608A	3.47	80A - 594A	3.954
32A - 596A	2.187	80A - 595A	3.246
33A - 596A	3.651	80A - 596A	3.02
33A - 598A	2.327	80A - 597A	4.093
33A - 599A	2.355	5B - 189A	3.709
33A - 610A	4.654	7B - 189A	2.168
34A - 599A	4.472	8B - 189A	3.466
34A - 607A	4.556	9B - 591A	3.824
34A - 608A	3.065	90B - 189A	1.124
34A - 609A	4.214	91B - 189A	4.899
34A - 610A	3.64	91B - 591A	3.696
35A - 598A	4.413	202A - 596A	4.819
35A - 599A	4.274	202A - 598A	3.925
35A - 602A	2.483	205A - 594A	3.001

c

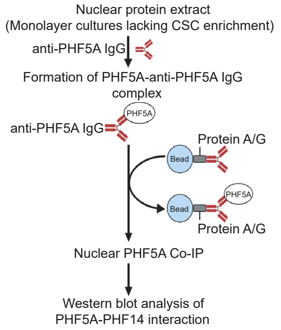

d

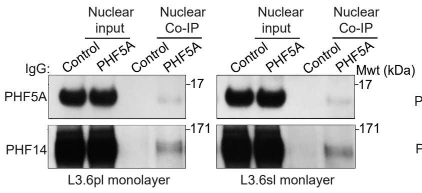

e

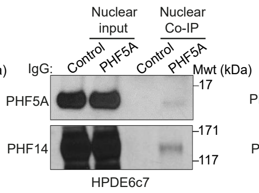

f

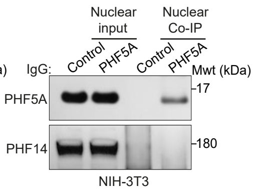

Supplementary Figure S2. Physical association between PHF5A and PHF14

a, Structure-based molecular modeling of the physical interaction between PHF5A (green) and PHF14 (orange), with interacting residues shown as sticks and the rest of the residues shown in the cartoon. Good hydrogen bonds (as determined by PyMOL) are shown in yellow. Electrostatic clashes (donor-donor or acceptor-acceptor) are shown in red. Close (<4.0 A) but not ideal contacts are shown in purple. Distances between interface residue pairs are labeled.

b, List of PHF5A-PHF14 interface residues within 5.0 Angstroms (A) between molecules and their corresponding distances in A.

c, Schematic illustration of PHF5A Co-IP from nuclear protein extracts of monolayer cultures lacking CSC enrichment for western blot analysis of the physical association between PHF5A and PHF14. The schematic illustration was created with Biorender scientific illustration software.

d-f, Western blot analysis of PHF5A Co-IP from nuclear protein extracts of monolayer cultures of L3.6pl, L3.6sl (**d**), HPDE6c7 (**e**), and NIH-3T3 (**f**). Data are representative of 2 biologically independent experiments per cell line with similar results.

Supplementary Figure S3

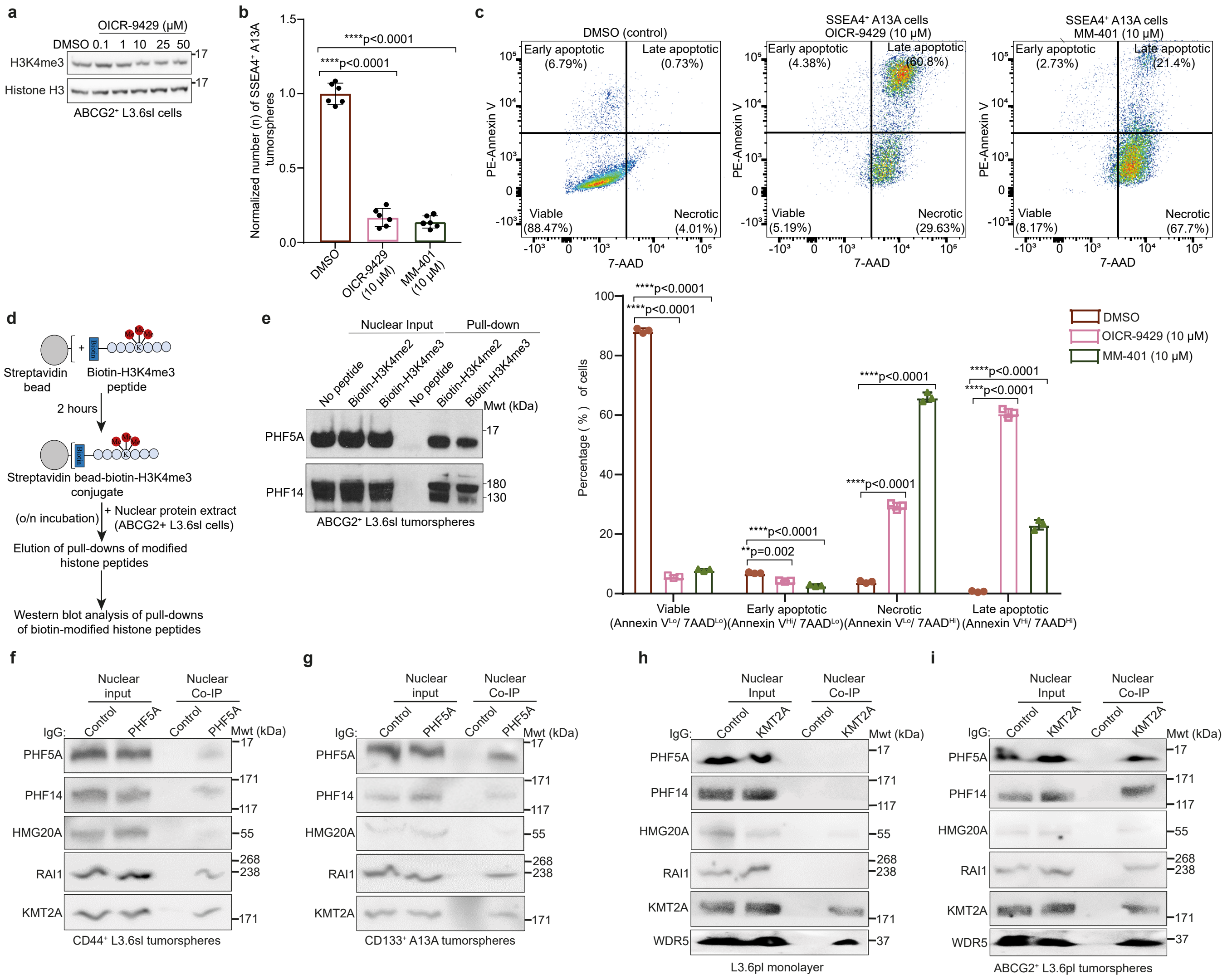

Supplementary Figure S3. KMT2A epigenetically regulates PCSCs and specifically associates with PHF5A-PHF14-HMG20A-RAI1-WDR5 protein subcomplex in the CSC population of cells

a, Western blot analysis of H3K4me3 protein levels in ABCG2$^+$ L3.6sl cells treated with different concentrations of OICR-9429 (0 – 50 µM) for 5 days. Total histone H3 was used as a loading control for extracted histone proteins. Data are representative of 2 biologically independent experiments with similar results.

b, Column chart demonstrating the effects of OICR-9429 and MM-401 on the sphere-forming capacity of SSEA4$^+$ A13A cells as compared to DMSO-treated (control) cells. Data are presented as the mean value ± SEM (n = 6 biologically independent experiments). Statistical analysis was performed using one-way ANOVA with multiple comparisons.

c, upper panel: Representative pseudocolor plots of flow cytometry analysis of cell viability and apoptosis following PE-Annexin V and 7-AAD staining of SSEA4$^+$ A13A cells treated with either DMSO, OICR-9429, or MM-401 for 5 days. **c, lower panel:** Graphical presentation of the percentages (%) of viable, early apoptotic, necrotic, late apoptotic, and necrotic SSEA4$^+$ A13A cells treated with either DMSO, OICR-9429, or MM-401 for 5 days, as determined by flow cytometry analysis of PE-Annexin V and 7-AAD-stained SSEA4$^+$ A13A cells. Data are presented as the mean value ± SEM (n = 6 biologically independent experiments). Statistical analysis was performed using two-way ANOVA with multiple comparisons.

d, Schematic illustration of biotin-H3K4me2 and biotin-H3k4me3 pull-down assays from nuclear protein extracts of ABCG2$^+$ L3.6sl cells. The schematic illustration was created with Biorender scientific illustration software.

e, Western blot analysis of eluates from biotin-H3K4me2 and biotin-H3K4me3 pull-downs from nuclear protein extracts of ABCG2$^+$ L3.6sl cells. Data are representative of 2 biologically independent experiments with similar results.

f and **g,** Western blot analysis of PHF5A Co-IP from nuclear protein extracts of CD44$^+$ L3.6sl (**f**) and CD133$^+$ A13A (**g**) tumorspheres.

h and **i,** Western blot analysis of KMT2A Co-IP from nuclear protein extracts of L3.6pl monolayer (**h**) and ABCG2$^+$ L3.6pl tumorspheres (**i**).

Supplementary Figure S4

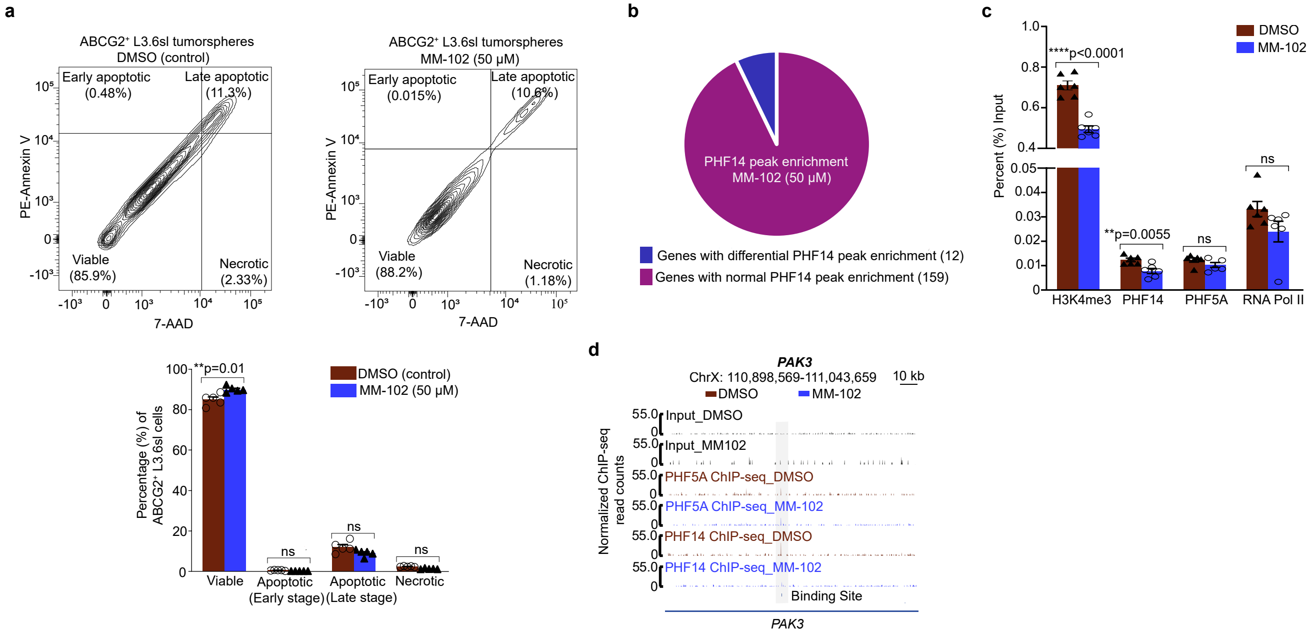

Supplementary Figure S4. KMT2A mediates the binding of PHF14 to target genomic sites

a, upper panel, Representative contour plots of flow cytometry analysis of apoptosis following PE-Annexin V and 7-AAD staining of ABCG2$^+$ L3.6sl cells treated with either DMSO (**left panel**) or 50 µM MM-102 (**right panel**) for 5 days. **a, lower panel,** Graphical presentation of the percentages (%) of viable, early apoptotic, late apoptotic, and necrotic ABCG2$^+$ L3.6sl cells treated with either DMSO or 50 µM MM-102 for 5 days as measured by flow cytometry analysis of PE-Annexin V and 7-AAD-stained cells. Data are presented as the mean value ± SEM (n = 5 biologically independent experiments). Statistical analysis was performed using a two-tailed *t* test with Welch's correction for unequal variances.

b, Pie chart illustrating the number of genes showing normal and differential PHF14 peak enrichment at target genomic sites in ABCG2$^+$ L3.6pl cells treated with 50 µM MM-102 versus DMSO (control) for 5 days.

c, ChIP-qPCR analysis of the differential enrichment for H3K4me3, PHF14, PHF5A, and RNA pol II peaks at *PAK3* target site in ABCG2$^+$ L3.6pl cells treated with MM-102 versus DMSO for 5 days. Data are presented as the mean value ± SEM (n = 6 biologically independent experiments). P values were calculated using a two-tailed *t* test with Welch's correction for unequal variances.

d, ChIP-seq track demonstrating the significant decrease in PHF14 peak enrichment at the target genomic site located within an intron of *PAK3* gene in ABCG2$^+$ L3.6pl cells treated with 50 µM MM-102 (blue tracks) versus DMSO (brown tracks) for 5 days. Differential binding analysis was performed using DiffBind v3.6.1, and peaks with adjusted p-value < 0.01 were considered significant.

Supplementary Figure S5

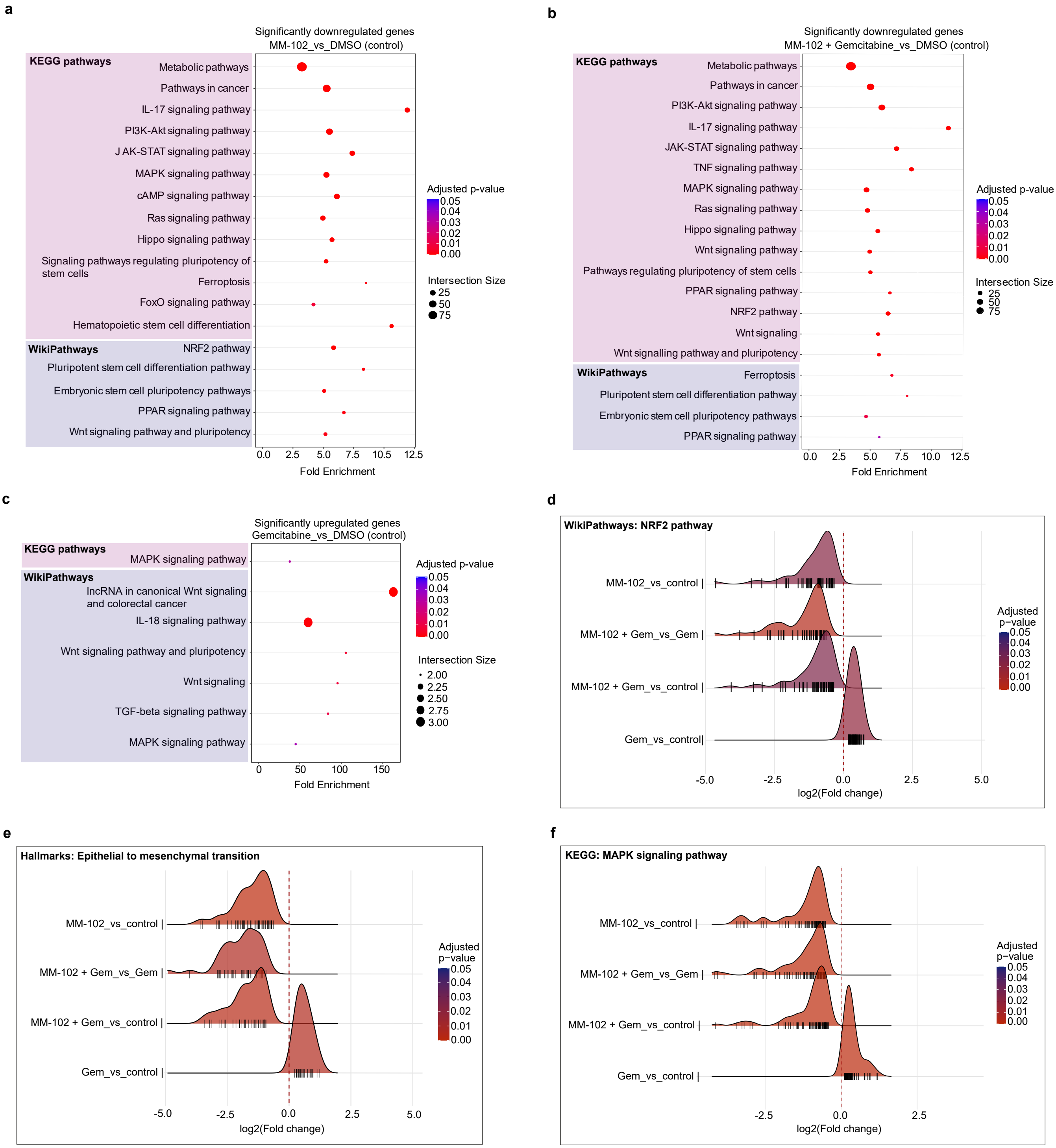

Supplementary Figure S5. RNA-seq analysis in MM-102 and gemcitabine-treated PCSCs

a-c, KEGG/wikiPathway enrichment analyses of significantly downregulated genes in MM-102 (**a**) and combined MM-102 and gemcitabine (**b**) treatment groups, and upregulated genes in the gemcitabine-treated group (**c**) as compared to the DMSO (control) treatment group. The size and color of the dots represent the number of genes enriched in the pathway of interest and the significance of enrichment, respectively. The statistical test was performed using the method provided by g:GOSt which uses the well-proven cumulative hypergeometric test. The multiple testing correction was performed using the default g:SCS (Set Counts and Sizes) correction method.

d-f, Ridge plots demonstrating the density distributions of log2 fold changes of enriched genes for GSEA enriched categories, including NRF2 pathway (WikiPathway, **d**), epithelial to mesenchymal transition (Hallmark, **e**), and MAPK signaling pathway (KEGG, **f**) in MM-102 versus control, MM-102 and gemcitabine versus gemcitabine, MM-102 and gemcitabine versus control, and gemcitabine versus control treatment groups. Upregualed and downregulated genes are indicated by positive and negative values, respectively, with colored peaks representing the adjusted p-value. Statistical test algorithms were provided by the package (fgsea used GSEA approach). The statistic (enrichment score, ES) is the weighted Kolmogorov–Smirnov statistic comparing the ranks of genes in G with the uniform distribution. Significance of the Q1 statistic is calculated by permutation of samples.

Supplementary Table 1. Library of small molecule compounds targeting epigenetic regulatory enzymes

	Compound name	Working concentration (µM)	Target
1	(+)-JQ1	1	BRD4(1/2)
2	(-)-JQ1 (inactive)	1	Bromodomains - Negative control
3	PFI-1	5	BRD4
4	I-BET762	1	BRD4
5	Bromosporine	1	Bromodomain and Extra-Terminal motif (BET)
6	CBP/BRD4 (0383)	5	Bromodomains - CBP, BRD4
7	SGC-CBP30	1	CBP/p300 bromodomain – BRD4
8	I-CBP112	1	CBP/p300 bromodomain
9	RVX-208	5	BET – BD1 and BD2
10	SMARCA	2.5	Bromodomains - SMARCA, PB1
11	PB1/SMARCA	1	Bromodomains - SMARCA, PB1
12	PFI-3	1	Bromodomains - SMARCA2/4
13	GSK2801	1	Bromodomains - BAZ2A, BAZ2B
14	PFI-4	1	Bromodomains - BRPF1B
15	TRIM24/BRPF	10	Bromodomains - TRIM24/BRPF
16	OF-1	5	Bromodomains - pan-BRPF
17	Belinostat	5	HDAC
18	CXD101	1	HDAC - HDAC1/2/3
19	Valproic acid	1000	HDAC – HDAC1/2
20	Entinostat	0.5	HDAC – HDAC1/2/3
21	SAHA	2.5	HDAC – HDAC1/2/3/6/7/11
22	Trichostatin A	0.5	HDAC – HDAC Class I & II
23	SRT1720	1	SirT1/2/3
24	EX-527	1	SirT1
25	CI-994	1	HDAC – HDAC1/2/3
26	CPI-360	10	Histone methyltransferase – EZH1
27	CPI-413	10	Histone methyltransferase - EZH2 and EZH1
28	UNC0638	1	Histone methyltransferase - G9a, GLP
29	UNC0642	1	Histone methyltransferase - G9a, GLP
30	A-366	2	Histone methyltransferase - G9a, GLP
31	Chaetocin	0.05	Histone methyltransferase - dSU(VAR)3-9, mouse G9a and Neurospora crassa DIM5
32	PFI-2	2	Lysine methyltransferase - SETD7
33	SGC0946	7.5	Histone methyltransferase - DOT1L
34	GSK343	3	Histone methyltransferase - EZH2
35	UNC1999	1	Histone methyltransferase – EZH1/2
36	LLY-507	1	Histone methyltransferase - SMYD2
37	Tranylcypromine	20	Monoamine oxidase – CY02A6
38	GSK-LSD1 (irreversible)	0.5	Lysine demethylases - LSD1
39	GSK690	5	Lysine demethylases - LSD1

40	GSK-J4	10	Lysine demethylases - JMJD3/KDM6B and UTX/KDM6A
41	GSK J5 hydrochloride	10	Negative control for GSK-J4
42	IOX1 (5-carboxy-8HQ)	40	Lysine demethylases - Broad-spectrum inhibitor of 2OG oxygenases
43	Methylstat	2.5	Histone demethylase
44	JIB-04	0.05	Histone demethylase - JARID1A, JMJD2E, JMJD3, JMJD2A, JMJD2B, JMJD2C, and JMJD2D,
45	ML324	5	Histone demethylase - JMJD2
46	IOX2	10	HIF modulator - HIF-1α prolyl hydroxylase-2 (PHD2)
47	OICR-9429	1	Histone methyl transferase - Antagonist of the interaction of WDR5 with peptide regions of MLL and Histone 3
48	UNC1215	5	Methyllysine (Kme) reading domain - L3MBTL3
49	5-Azacitidine	10	DNA methyltransferase (DNMT)
50	5-Azadeoxycitidine	5	DNA methyltransferase (DNMT) - DNMT1/3
51	Olaparib	1	Poly ADP ribose polymerase (PARP) – PARP1/2
52	Rucaparib	10	Poly ADP ribose polymerase (PARP) – PARP1/2/3
53	K00135	1	Adenosine kinase - ATP competitive - PIM
54	5-Iodotubercidin	1	Kinase inhibitor - CK1, insulin receptor tyrosine kinase, phosphorylase kinase, PKA, CK2, PKC and Haspin.
55	C646	1	Histone acetyltransferase p300
56	DUAL1946	1	
57	GSK484	1	Peptidylarginine deiminase (PAD) – PAD4
58	KDOBA67	10	Histone demethylase
59	BAZ2-ICR	1	Bromodomains - BAZ2A/B
60	NI-57	1	Bromodomain and plant homeodomain finger-containing (BRPF) - BRPF1, BRPF2 (BRD1) and BRPF3
61	LP99	1	Bromodomains – BRD7 and BRD9
62	SGC707	1	Histone methyltransferase - PRMT3
63	RGFP966	10	HDAC - HDAC3
64	PCI-34051	5	HDAC - HDAC8
65	Rocilinostat	10	HDAC - HDAC6 (potent),<br>also inhibits HDAC1, HDAC2 and HDAC3
66	Tubastatin A HCl	10	HDAC - HDAC6 (potent), also inhibits HDAC8, HDAC10 and metallo-β-lactamase domain-containing protein 2 (MBLAC2)
67	KDOAM-25	1	Histone demethylases – KDM5A/B/C/D
68	KDM5-C70	10	Histone demethylase – pan-KDM5 histone demethylase
69	MAZ1805	1	t-RNA sythetase
70	MAZ1392	1	t-RNA sythetase
71	BI-9564	1	Bromodomains - BRD9/BRD7
72	NVS-CECR2-1	1	Non-BET family Bromodomain (BRD) – CECR2

73	GSK106	1	Protein arginine deiminase - Inactive control for the selective PAD4 inhibitors, GSK484 and GSK199.
74	J556-42R	1	Protein arginine methyltransferase - PRMT5
75	J556-63R	1	Protein arginine methyltransferase - PRMT5
76	J556-70R	1	Protein arginine methyltransferase - PRMT5
77	A-196	1	Histone methyltransferase - SUV420H1 and SUV420H2
78	BAY-598	1	Histone methyltransferase - SMYD2
79	J556-143	1	Protein arginine methyltransferase - PRMT5
80	MS049	1	Protein arginine methyltransferase – PRMT4/6
81	MS023	1	Protein arginine methyltransferase - Type I PRMTs
82	MS003	1	Protein arginine methyltransferase - negative control
83	SGI-1776	10	Pim kinases – Pim-1/2/3
84	CHR-6494	1	Haspin kinase - Haspin
85	CPI-169	10	Histone methyltransferase - EZH2 WT, EZH2 Y641N, and EZH1
86	UNC2400	1	Negative control for Histone methyltransferase
87	GSK864	5	Isocitrate Dehydrogenase - IDH1 mutants R132C, R132H, and R132G
88	GSK8814	10	Bromodomains - ATAD2/2B
89	GSK8815	10	Bromodomains - ATAD2
90	GSK959	1	Bromodomains - BRPF1 bromodomain
91	NVS-CECR2-C	1	Non-BET family Bromodomain inhibitor - CECR2
92	BAY-299	1	BRPF2 bromodomains - TAF1 BD2 and TAF1L BD2.
93	PCI-24781	10	HDAC - pan-HDAC
94	Romidepsin	1	HDAC - HDAC1/2/4/6
95	Mocetinostat	10	HDAC - HDAC1/2/3/11
96	Santacruzamate A	50	HDAC - HDAC2
97	KDOAM32	1	Lysine demethylases - JARID
98	MS409N	1	Negative control for Protein arginine methyltransferase - PRMT4/6
99	TP-064	1	Protein arginine methyltransferase - PRMT4
100	TP-064N	1	Protein arginine methyltransferase - PRMT4
101	A-395	1	Histone methyltransferase - Trimeric PRC2 complex (EZH2-EED-SUZ12)
102	A-395N	1	Negative control for A-395
103	I-BRD9	10	Bromodomains - BRD9
104	TP-472	1	Bromodomains – BRD7/9
105	TP-472N	1	Negative control for TP-472
106	KDOPZ-32a	1	Lysine demethylases - KDM5
107	KDOOA012000	1	Lysine demethylases KDM2
108	AMI-1	50	Protein arginine methyltransferase – PRMT1
109	TMP269	10	HDAC - HDAC4/5/7/9

110	AGK2	10	SIRT2 (selective), also inhibits SIRTT1/3 at higher IC_{50}
111	GSK6853	1	Bromodomains - BRPF1
112	GSK9311	1	Bromodomains - BRPF1/2
113	LLY-283	1	Protein arginine methyltransferase - PRMT5
114	TD001851a	1	Methyl Lysine Binder/tudor domain -Spin1
115	TDOSI000058a	1	Methyl Lysine Binder/tudor domain -Spin1
116	TD001863a	1	Methyl Lysine Binder/tudor domain -Spin1
117	TDOSI000062a	1	Methyl Lysine Binder/tudor domain -Spin1
118	TD001857a	1	Methyl Lysine Binder/tudor domain -Spin1
119	TD001856a	1	Methyl Lysine Binder/tudor domain -Spin1
120	TD001858a	1	Methyl Lysine Binder/tudor domain -Spin1
121	TMP195	1	HDAC - HDAC4/5/7/9
122	GSK2879552	10	Histone demethylase – LSD1/KDM1A
123	TDO20821a	1	Methyl Lysine Binder/tudor domain -Spin1
124	TDO20824a	1	Methyl Lysine Binder/tudor domain -Spin1
125	TDO20823a	1	Methyl Lysine Binder/tudor domain -Spin1
126	A-485	1	Histone acetyltransferase (HAT) p300/CBP
127	A-486	1	Histone acetyltransferase (HAT) p300/CBP
128	GSK4027	1	Bromodomains – PCAF/GCN5
129	GSK4028	1	Negative control for GSK4027
130	L-Moses	1	Bromodomains - PCAF, GCN5
131	D-Moses	1	Bromodomains - PCAF, GCN5
132	PFI-5	1	Histone methyltransferase - SMYD2
133	YX39-31b	1	Methyl Lysine Binder/tudor domain -Spin1
134	TDO208229	1	Methyl Lysine Binder/tudor domain -Spin1
135	TDO01856a	1	Methyl Lysine Binder/tudor domain -Spin1
136	TDO20826a	1	Methyl Lysine Binder/tudor domain -Spin1
137	Bortezomib	0.1	Proteasome
138	Carfilzomib	0.1	Proteasome
139	RTS-V5	1	Proteasome and HDAC
140	dBRD9	1	Bromodomains - BRD9
141	BI-7273	0.1	Bromodomains - BRD9/7
142	CPI-621	0.1	Lysine demethylases - KDM5

Supplementary Table 2. Sequences of primers used in qRT-PCR amplification

Gene	Forward primer sequence (5’-3’)	Reverse primer sequence (5’-3’)
SOX2	ATGAATGCCTTCATGGTGTGG	CGGTATTTATAATCCGGGTGCT
NANOG	GACTGTCTCTCCTCTTCCTTC	CTGGTCTTCTGTTTCTTGACC
KLF4	ACCCACACAGGTGAGAAACC	ATGTGTAAGGCGAGGTGGTC
STAT3	ATGGCCCAATGGAATCAGC	CCGCATATGCCCAATCTTG
POU5F1	CGACCATCTGCCGCTTTG	CTGCTTTGCATATCTCCTGAAG
ACTB	CACCAGGGCGTGATGGTG	GAGCCACACGCAGCTCAT
