# Supplementary material for: KMT2A associates with PHF5A-PHF14-HMG20A-RAI1 subcomplex in pancreatic cancer stem cells and epigenetically regulates their characteristics
Source: Nat Commun. 2023 Sep 14;14:5685. doi: 10.1038/s41467-023-41297-4 (PMC10502114; doi:10.1038/s41467-023-41297-4)
Supplement: Supplementary file 3 — Reporting Summary [file 41467_2023_41297_MOESM3_ESM.pdf]

nature portfolio

Corresponding author(s): Siim Pauklin

Last updated by author(s): 13/08/2023

Reporting Summary

Nature Portfolio wishes to improve the reproducibility of the work that we publish. This form provides structure for consistency and transparency in reporting. For further information on Nature Portfolio policies, see our Editorial Policies and the Editorial Policy Checklist.

Statistics

For all statistical analyses, confirm that the following items are present in the figure legend, table legend, main text, or Methods section.

n/a	Confirmed	
☐	☒	The exact sample size (*n*) for each experimental group/condition, given as a discrete number and unit of measurement
☐	☒	A statement on whether measurements were taken from distinct samples or whether the same sample was measured repeatedly
☐	☒	The statistical test(s) used AND whether they are one- or two-sided<br>*Only common tests should be described solely by name; describe more complex techniques in the Methods section.*
☒	☐	A description of all covariates tested
☐	☒	A description of any assumptions or corrections, such as tests of normality and adjustment for multiple comparisons
☐	☒	A full description of the statistical parameters including central tendency (e.g. means) or other basic estimates (e.g. regression coefficient) AND variation (e.g. standard deviation) or associated estimates of uncertainty (e.g. confidence intervals)
☐	☒	For null hypothesis testing, the test statistic (e.g. *F*, *t*, *r*) with confidence intervals, effect sizes, degrees of freedom and *P* value noted<br>*Give P values as exact values whenever suitable.*
☒	☐	For Bayesian analysis, information on the choice of priors and Markov chain Monte Carlo settings
☒	☐	For hierarchical and complex designs, identification of the appropriate level for tests and full reporting of outcomes
☒	☐	Estimates of effect sizes (e.g. Cohen's *d*, Pearson's *r*), indicating how they were calculated

Our web collection on statistics for biologists contains articles on many of the points above.

Software and code

Policy information about availability of computer code

Data collection	- Flow cytometry data acquisition was performed using BD LSRFortessa cell analyzer (BD Biosciences) and collected with FACSDiva Software (BD, Version 8.0.1).<br>- Quantitative real-time PCR data were acquired using a ViiA7 Real-time PCR system (Applied Biosystems) with QuantStudio Software v1.6.1 (Applied Biosystems) and LightCycler 480 System (Roche).<br>- Tumorspheres data acquisition, including spheres count and imaging, was performed using Celigo Imaging Cytometer (Nexcelom Bioscience) with Celigo Software (Version 2.1).<br>- ChIP-seq and RNA-seq data were acquired using Illumina NovaSeq6000 S4. BCL convert v4.0.3 was used to generate the fastq raw data.<br>- A FluoStar Omega microplate reader (BMG Labtech) was used to measure the number of live cells stained with PrestoBlue cell viability reagent (Thermo Fisher Scientific, A13261) as per the manufacturer's instructions.
Data analysis	Publicly available softwares: FlowJo v10.8; Scaffold (version Scaffold_4.10.0); fastp v0.23.2; FastQC v0.11; Burrows-Wheeler Aligner v0.7.17; MACS2 v2.2.7.1; ChIPpeakAnno v3.30.0; DiffBind v3.6.1; STAR v2.7.3a; featureCounts v2.0.0; DESeq2 v1.34.0; fgsea v1.22.0; Molecular Signatures Database v7.5.1; g:GOSt; HDOCK; GraphPad Prism 8; and DNA Dynamo (Version 1.0).

For manuscripts utilizing custom algorithms or software that are central to the research but not yet described in published literature, software must be made available to editors and reviewers. We strongly encourage code deposition in a community repository (e.g. GitHub). See the Nature Portfolio guidelines for submitting code & software for further information.

Data

Policy information about availability of data

All manuscripts must include a data availability statement. This statement should provide the following information, where applicable:

- Accession codes, unique identifiers, or web links for publicly available datasets
- A description of any restrictions on data availability
- For clinical datasets or third party data, please ensure that the statement adheres to our policy

Raw and processed ChIP-seq and RNA-seq data (BioProject PRJNA887833; https://www.ncbi.nlm.nih.gov/bioproject/PRJNA887833) are available in the Sequence Read Archive (SRA) under accession code SRP403939 (https://www.ncbi.nlm.nih.gov/sra/?term=SRP403939) and the Gene Expression Ominbus (GEO) under accession code GSE217332 (https://www.ncbi.nlm.nih.gov/geo/query/acc.cgi?acc=GSE217332), with no restrictions on data availability. The mass spectrometry proteomics data have been deposited to the ProteomeXchange Consortium via the PRIDE [1] partner repository with the dataset identifier PXD038378 (Project Webpage: http://www.ebi.ac.uk/pride/archive/projects/PXD038378; FTP Download: ftp://ftp.pride.ebi.ac.uk/pride/data/archive/2023/08/PXD038378). The remaining data are available within the Article, Supplementary Information or Source Data file.

Human research participants

Policy information about studies involving human research participants and Sex and Gender in Research.

Reporting on sex and gender	The study did not involve human participants.
Population characteristics	The study did not involve human participants.
Recruitment	The study did not involve human participants.
Ethics oversight	The study did not involve human participants.

Note that full information on the approval of the study protocol must also be provided in the manuscript.

Field-specific reporting

Please select the one below that is the best fit for your research. If you are not sure, read the appropriate sections before making your selection.

☒ Life sciences ☐ Behavioural & social sciences ☐ Ecological, evolutionary & environmental sciences

For a reference copy of the document with all sections, see nature.com/documents/nr-reporting-summary-flat.pdf

Life sciences study design

All studies must disclose on these points even when the disclosure is negative.

Sample size	No statistical methods were used to predetermine the sample size for in vitro and in vivo experiments, but preliminary experiments were conducted to determine the appropriate sample size that would provide sufficient statistical power. For in vitro experiments, 3-6 biological replicates were used, which allowed us to perform statistical analysis using two-tailed Student t test with Welch's correction in case of significantly different variances as analyzed with F-test and one/two-way ANOVA with multiple comparisons with Tukey correction. For in vivo experiments, sample size was determined based on pilot studies performed in athymic mice to test the in vivo tumorigenicity of ABCG2+ L3.6pl cells and determine the maximal tolerated dose (MTD) of MM-102. In our reported experiments (Fig. 6l-n), 6 mice were used per each treatment group, and which we found sufficient to account for the biological variation observed in tumor growth and response to treatment in vivo. Sample sizes are indicated in the figure legends.
Data exclusions	No data were excluded from the study.
Replication	3-6 independent sample replicates were used for each experiment as indicated in the figure legends. All attempts at replication were successful.
Randomization	- Mice were first randomized into 3 treatment groups (6 mice per treatment group, 3 mice per cage) prior to subcutaneous inoculation of pancreatic cancer stem cells.<br>- For the subcutaneous tumor xenograft experiments, tumor-bearing mice were randomized again before being allocated to treatment groups, including vehicle, MM-102 (30 mg/kg/day), and MM-102 (50 mg/kg/day).<br>- For in vitro functional assays and RNA-seq analysis requiring drug treatment, pancreatic cancer stem cells were randomized for vehicle and drug treatments.<br>- For all other in vitro experiments including mass spectrometry, RT-qPCR, Co-IP and western blotting, histone peptides pull down assay, ChIP-seq, and small molecule compound screening, samples were randomized into the indicated groups.
Blinding	- Mass Spectrometry analysis was performed at the Cambridge Centre for Proteomics (CCP), where codes have been used to identify samples. Data were acquired by one researcher at the CCP facility and analyzed by a different researcher in a blinded manner.

- ChIP-seq and RNA-seq data acquisition was carried out at CD Genomics, USA, using sample codes to identify submitted samples, and data were analyzed by an independent bioinformatician.
- Blinding was not possible for all other data based on quantitative analysis during the conductance of the experiments for appropriate treatments to the assigned groups. However, the investigators and authors have been consistently blinded to the group allocation during data collection and analysis

Reporting for specific materials, systems and methods

We require information from authors about some types of materials, experimental systems and methods used in many studies. Here, indicate whether each material, system or method listed is relevant to your study. If you are not sure if a list item applies to your research, read the appropriate section before selecting a response.

Materials & experimental systems

n/a	Involved in the study
☐	☒ Antibodies
☐	☒ Eukaryotic cell lines
☒	☐ Palaeontology and archaeology
☐	☒ Animals and other organisms
☒	☐ Clinical data
☒	☐ Dual use research of concern

Methods

n/a	Involved in the study
☐	☒ ChIP-seq
☐	☒ Flow cytometry
☒	☐ MRI-based neuroimaging

Antibodies

Antibodies used

For Flow Cytometry: PerCP/Cy5.5 anti-ABCG2 (BioLegend, 332024; 1:50), APC anti-EPCAM (BioLegend, 324208; 1:50), PE anti-CD44 (BD Biosciences, 555479; 1:10), BV421 anti-CD24 (BD Biosciences, 562789; 1:50), BV786 anti-PROM1 (BD Biosciences, 747640; 1:50), FITC anti-SSEA4 (BD Biosciences, 560126; 1:10),BV510 anti-CXCR4 (BioLegend, 306535; 1:50), PerCP-Cy5.5 mouse IgG2b, k (BD Biosciences, 558020; 1:50), APC mouse IgG2b, k (BioLegend, 402206; 1:50), PE mouse IgG2a, k (BD Biosciences, 555574; 1:10), BV421 mouse IgG2a, k (BD Biosciences, 562439; 1:50), BV786 mouse IgG1, k (BD Biosciences, 563330; 1:50), FITC mouse IgG3, k (BD Biosciences, 556658; 1:10), and BV510 mouse IgG2a, k (BioLegend, 400267; 1:50), Pacific Blue anti-SOX2 antibody (BioLegend, 656111; 1:20), Alexa Fluor 647 anti-SSEA4 (BD Biosciences, 560796; 1:40) and its isotype control Alexa Fluor 647 Mouse IgG3, k (BD Biosciences, 560803; 1:40).
For MACS: Magnetic microbeads conjugated with anti-ABCG2 (Miltenyi Biotec, 130-107-680; 1:5) and anti-SSEA4 (Miltenyi Biotec, 130-097-855; 1:5).
For Co-immunoprecipitation: anti-SMAD2/3 (R and D Systems, AF3797; 1:200), anti-PHF5A (Proteintech, 15554-1-AP; 1:80), anti-PHF14 (Proteintech, 24787-1-AP; 1:260), anti-KMT2A (Abcam, ab272023; 1:200), normal goat IgG (R and D Systems, AB-108-C; 1:200), and rabbit (DA1E) mAb IgG XP (Cell Signaling Technology, 3900S; 1:500).
For Western blotting: SMAD2/3 (Cell Signaling Technology, 3102S; 1:2000), p-SMAD2 (Ser465/Ser467) (Thermo Fisher Scientific, 44-244G; 1:1000), p-SMAD3 (Ser423/Ser425) (Abcam, ab52903; 1:2000), SMAD4 (Proteintech, 10231-1-AP; 1:2000), PHF5A (Proteintech, 15554-1-AP; 1:2000), PHF14 (Proteintech, 24787-1-AP; 1:2000), α-Tubulin (Proteintech, 66031-1-Ig; 1:6000), Lamin B1 (Proteintech, 12987-1-AP; 1:2000), HMG20A (Proteintech, 12085-2-AP; 1:2000), RAI1 (Abcam, ab86599; 1:2000), TCF20 (Novus Biologicals, NBP2-83631; 1:1000), KMT2A (Proteintech, 29278-1-AP; 1:1000), RNA polymerase II CTD repeat YSPTSPS (Abcam, ab26721; 1:2000), H3K4me2 (Abcam, cat. ab32356, dilution 1:2000), H3K4me3 (Abcam, ab213224; 1:2000), Histone H3 (Abcam, ab1791; 1:6000), WDR5 (Proteintech, 15544-1-AP; 1:2000), anti-rabbit (Sigma-Aldrich, A0545; 1:10,000) or anti-mouse (Sigma-Aldrich, A9044; 1:10,000) horseradish peroxidase (HRP)-conjugated secondary antibodies.
For ChIP-seq: anti-PHF5A (Proteintech, 15554-1-AP; 1:200), anti-PHF14 (Proteintech, 24787-1-AP; 1:200), and anti-KMT2A (Proteintech, 29278-1-AP; 1:200).

Validation

All antibodies were commercially validated (see manufacturer's website link below), previously published or validated in our study.
Pacific Blue anti-SOX2 (BioLegend, cat. 656111), https://www.biolegend.com/en-gb/search-results/pacific-blue-anti-sox2-antibody-12471?GroupID=GROUP26.
Anti-SMAD2/3 (Cell Signaling Technology, cat. 3102S), https://www.cellsignal.com/products/primary-antibodies/smad2-3-antibody/3102
Anti-phospho-SMAD2 (Ser465/Ser467) (Thermo Fisher Scientific, cat. 44-244G), https://www.thermofisher.com/antibody/product/Phospho-SMAD2-Ser465-Ser467-Antibody-Polyclonal/44-244G
Anti-phospho-SMAD3 (Ser423/Ser425) (Abcam, ab52903), https://www.thermofisher.com/antibody/product/Phospho-SMAD3-Ser423-Ser425-Antibody-Polyclonal/44-246G.
Anti-SMAD4 (Proteintech, 10231-1-AP), https://www.ptglab.com/products/SMAD4-Antibody-10231-1-AP.htm.
Anti-PHF5A (Proteintech, cat. 15554-1-AP), https://www.ptglab.com/Products/PHF5A-Antibody-15554-1-AP.htm
Anti-PHF14 (Proteintech, cat. 24787-1-AP), https://www.ptglab.com/Products/PHF14-Antibody-24787-1-AP.htm
Anti-alpha Tubulin (Proteintech, cat. 66031-1-Ig), https://www.ptglab.com/products/tubulin-Alpha-Antibody-66031-1-Ig.htm
Anti-Lamin B1 (Proteintech, cat. 12987-1-AP), https://www.ptglab.com/products/LMNB1-Antibody-12987-1-AP.htm
Anti-HMG20A (Proteintech, cat. 12085-2-AP), https://www.ptglab.com/Products/HMG20A-Antibody-12085-2-AP.htm
Anti-RAI1 (Abcam, cat. ab86599), https://www.abcam.com/RAI1-antibody-ab86599.html
Anti-TCF20 (Novus Biologicals, cat. NBP2-83631), https://www.novusbio.com/products/tcf20-antibody_nbp2-83631
Anti-KMT2A (Proteintech, cat. 29278-1-AP), https://www.ptglab.com/products/MLL-Antibody-29278-1-AP.htm
Anti-RNA polymerase II CTD repeat YSPTSPS (RRID:AB_777726, Abcam, cat. ab2672), https://www.abcam.com/Anti-RNA-polymerase-II-CTD-repeat-YSPTSPS-antibody-ChIP-Grade-ab26721.html

Anti-H3K4me3 (Abcam, cat. ab213224), https://www.abcam.com/histone-h3-tri-methyl-k4-antibody-epr20551-225-chip-grade-ab213224.html
Anti-Histone H3 (Abcam, cat. ab1791), https://www.abcam.com/histone-h3-antibody-nuclear-marker-and-chip-grade-ab1791.html
Anti-rabbit HRP-conjugated secondary antibody (Sigma-Aldrich, cat. A0545) , https://www.sigmaaldrich.com/EG/en/product/sigma/a0545
Anti-WDR5 (Proteintech, cat. 15544-1-AP), https://www.ptglab.com/Products/WDR5-Antibody-15544-1-AP.htm
Anti-mouse HRP-conjugated secondary antibody (Sigma-Aldrich, cat. A9044), https://www.sigmaaldrich.com/EG/en/product/sigma/a9044)
Anti-ABCG2 magnetic microbeads (Miltenyi Biotec, cat. 130-107-680), https://www.miltenyibiotec.com/US-en/products/anti-abcg2-cd338-microbeads-human.html
Anti-SSEA4 magnetic microbeads (Miltenyi Biotec, cat. 130-097-855), https://www.miltenyibiotec.com/US-en/products/anti-ssea-4-microbeads-human.html
PerCP/Cy5.5 anti-ABCG2 (BioLegend, cat. 332024), https://www.biolegend.com/en-us/products/percp-cyanine5-5-anti-human-cd20-antibody-4228?GroupID=BLG7904
APC anti-EPCAM (BioLegend, cat. 324208), https://www.biolegend.com/en-us/products/apc-anti-mouse-cd326-ep-cam-antibody-4974?GroupID=BLG5748
PE anti-CD44 (BD Biosciences, cat. 555479), https://www.bdbiosciences.com/en-us/products/reagents/flow-cytometry-reagents/research-reagents/single-color-antibodies-ruo/pe-mouse-anti-human-cd44.555479
BV421 anti-CD24 (BD Biosciences, cat. 562789), https://www.bdbiosciences.com/en-us/products/reagents/flow-cytometry-reagents/research-reagents/single-color-antibodies-ruo/bv421-mouse-anti-human-cd24.562789
BV786 anti-PROM1 (BD Biosciences, cat. 747640), https://www.bdbiosciences.com/en-us/products/reagents/flow-cytometry-reagents/research-reagents/single-color-antibodies-ruo/bv786-mouse-anti-human-cd133.747640
FITC anti-SSEA4 (BD Biosciences, cat. 560126), https://www.bdbiosciences.com/en-us/products/reagents/flow-cytometry-reagents/research-reagents/single-color-antibodies-ruo/fitc-mouse-anti-ssea-4.560126
BV510 anti-CXCR4 (BioLegend, cat. 306535), https://punchout.biolegend.com/en-us/search-results/brilliant-violet-510-anti-human-cd184-cxcr4-antibody-17653?GroupID=BLG8071
PerCP-Cy5.5 mouse IgG2b, k (BD Biosciences, cat. 558020), https://www.bdbiosciences.com/en-us/products/reagents/flow-cytometry-reagents/research-reagents/flow-cytometry-controls-and-lysates/percp-cy-5-5-mouse-igg2a-isotype-control.558020
APC mouse IgG2b, k (BioLegend, cat. 402206), https://www.biolegend.com/en-us/products/apc-mouse-igg2b-kappa-isotype-ctrl-14793
PE mouse IgG2a, k (BD Biosciences, cat. 555574), https://www.bdbiosciences.com/en-us/products/reagents/flow-cytometry-reagents/research-reagents/flow-cytometry-controls-and-lysates/pe-mouse-igg2a-isotype-control.555574
BV421 mouse IgG2a, k (BD Biosciences, cat. 562439), https://www.bdbiosciences.com/en-us/products/reagents/flow-cytometry-reagents/research-reagents/flow-cytometry-controls-and-lysates/bv421-mouse-igg2a-k-isotype-control.562439
BV786 mouse IgG1, k (BD Biosciences, cat. 563330), https://www.bdbiosciences.com/en-us/products/reagents/flow-cytometry-reagents/research-reagents/flow-cytometry-controls-and-lysates/bv786-mouse-igg1-k-isotype-control.563330
FITC mouse IgG3, k (BD Biosciences, cat. 556658), https://www.bdbiosciences.com/en-us/products/reagents/flow-cytometry-reagents/research-reagents/flow-cytometry-controls-and-lysates/fitc-mouse-igg3-isotype-control.556658
BV510 mouse IgG2a, k (BioLegend, cat. 400267), https://www.biolegend.com/en-us/products/brilliant-violet-510-mouse-igg2a-kappa-isotype-ctrl-8015

Eukaryotic cell lines

Policy information about cell lines and Sex and Gender in Research

Cell line source(s)

L3.6pl, L3.6sl, and FG pancreatic cancer cell lines were purchased from MD Anderson Cancer Center (USA). A13A and A1 3B pancreatic cancer cell lines were kindly gifted by Professor Christine Iacobuzio-Donahue (Memorial Sloan Kettering Cancer Center, USA) in 2018 and 2020, respectively. The immortalized human pancreatic ductal epithelial cell line HPDE6c7 was purchased from Kerafast, cat. no ECA001-FP (USA). NIH-3T3 cell line was kindly gifted by Prof. Udo Oppermann (Botnar Research Centre, originally purchased from ATCC, cat. no CRL-1658).

Authentication

L3.6pl, L3.6sl, and FG cell lines were authenticated using Short Tandem Repeat DNA profiling. We have not performed additional authentication for A13A, A13B, NIH-3T3, or HPDE6c7 cell lines (Previously authenticated by Short Tandem Repeat DNA profiling).

Mycoplasma contamination

A PCR-based method for the detection of Mycoplasma contamination in cell cultures was routinely performed using the following primer sequences: forward primer; 5’-GGGAGCAAACAGGATTAGATACCCT-3’ and reverse primer; 5’-TGCACCATCTGTCACTCTGTTAACCTC-3’. All cell lines tested negative for mycoplasma contamination.

Commonly misidentified lines
(See ICLAC register)

No commonly misidentified cell lines were used in this study.

Animals and other research organisms

Policy information about studies involving animals; ARRIVE guidelines recommended for reporting animal research, and Sex and Gender in Research

Laboratory animals

6-week old female athymic nude (Foxn1nu) mice (RRID: IMSR_JAX:002019) were purchased from the Jackson laboratory. Female mice were only used as as the use of males and females in a same study could reduce statistical power because of a greater spread of pooled data or smaller sub-samples of each sex.
Animals were housed in a pathogen-free environment under ambient temperature and standard light-dark cycle conditions at the

	Genome and Biomedical facility at the University of California Davis (USA). The study received ethical approval from the Institutional Animal Care and Use Committee (IACUC), Protocol number 22444. The animal sex was not selected for a specific reason as sex is not a variable factor in our study.
Wild animals	The study did not involve wild animals.
Reporting on sex	This information has not been collected, as it is irrelevant to our study.
Field-collected samples	The study did not involve Field-collected samples.
Ethics oversight	All experiments were conducted on mice, housed in a pathogen-free environment under ambient temperature and standard light-dark cycle conditions at the Genome and Biomedical facility at the University of California Davis, in accordance with procedures approved by the Institutional Animal Care and Use Committee (IACUC). In our experiments, the maximum tumor size at the largest diameter was 12.5 mm which is lower than the maximum tumor size permitted by IACUC (20 mm). Method used for euthanasia was carbon dioxide inhalation which involved placing conscious mice in chambers that were gradually filled with 100% carbon dioxide, then left for at least 3 minutes before resection of subcutaneous tumors.

Note that full information on the approval of the study protocol must also be provided in the manuscript.

ChIP-seq

Data deposition

☒ Confirm that both raw and final processed data have been deposited in a public database such as GEO.

☒ Confirm that you have deposited or provided access to graph files (e.g. BED files) for the called peaks.

Data access links
May remain private before publication.

Raw data (deposited in SRA): https://dataview.ncbi.nlm.nih.gov/object/PRJNA887833?reviewer=k77u9hufvcnef9kalq98ddjtrl
Processed data (deposited in GEO): Go to https://www.ncbi.nlm.nih.gov/geo/query/acc.cgi?acc=GSE217332 and enter the private secure token yhgvmquodrkplkj into the box.
Protein mass spectrometry data including raw and mascot dat files are available in the ProteomeXchange repository.

Files in database submission

Raw data (deposited in SRA):
input_KMT2A_rep_1_R1.fastq.gz input_KMT2A_rep_1_R2.fastq.gz
input_PHF5A_PHF14_rep_1_R1.fastq.gz input_PHF5A_PHF14_rep_1_R2.fastq.gz
KMT2A_rep_2_R1.fastq.gz KMT2A_rep_2_R2.fastq.gz
KMT2A_rep_3_R1.fastq.gz KMT2A_rep_3_R2.fastq.gz
PHF14_rep_1_R1.fastq.gz PHF14_rep_1_R2.fastq.gz
PHF14_rep_2_R1.fastq.gz PHF14_rep_2_R2.fastq.gz
PHF5A_rep_1_R1.fastq.gz PHF5A_rep_1_R2.fastq.gz
PHF5A_rep_2_R1.fastq.gz PHF5A_rep_2_R2.fastq.gz

input_PHF5A_PHF14_Ctrl_rep_1_R1.fastq.gz input_PHF5A_PHF14_Ctrl_rep_1_R2.fastq.gz
input_PHF5A_PHF14_MM102_rep_1_R1.fastq.gz input_PHF5A_PHF14_MM102_rep_1_R2.fastq.gz
PHF14_Ctrl_rep_2_R1.fastq.gz PHF14_Ctrl_rep_2_R2.fastq.gz
PHF14_Ctrl_rep_3_R1.fastq.gz PHF14_Ctrl_rep_3_R2.fastq.gz
PHF14_MM102_rep_1_R1.fastq.gz PHF14_MM102_rep_1_R2.fastq.gz
PHF14_MM102_rep_2_R1.fastq.gz PHF14_MM102_rep_2_R2.fastq.gz
PHF5A_Ctrl_rep_2_R1.fastq.gz PHF5A_Ctrl_rep_2_R2.fastq.gz
PHF5A_Ctrl_rep_3_R1.fastq.gz PHF5A_Ctrl_rep_3_R2.fastq.gz
PHF5A_MM102_rep_2_R1.fastq.gz PHF5A_MM102_rep_2_R2.fastq.gz
PHF5A_MM102_rep_3_R1.fastq.gz PHF5A_MM102_rep_3_R2.fastq.gz

Processed data (deposited in GEO):
input_KMT2A_rep_1.bw
input_PHF5A_PHF14_rep_1.bw
KMT2A_rep_2.bw
KMT2A_rep_3.bw
PHF14_rep_1.bw
PHF14_rep_2.bw
PHF5A_rep_1.bw
PHF5A_rep_2.bw

input_PHF5A_PHF14_Ctrl_rep_1.bw
input_PHF5A_PHF14_MM102_rep_1.bw
PHF14_Ctrl_rep_2.bw

PHF14_Ctrl_rep_3.bw
PHF14_MM102_rep_1.bw
PHF14_MM102_rep_2.bw
PHF5A_Ctrl_rep_2.bw
PHF5A_Ctrl_rep_3.bw
PHF5A_MM102_rep_2.bw
PHF5A_MM102_rep_3.bw

Genome browser session (e.g. UCSC)

https://genome.ucsc.edu/s/siwei/Mai_ChIP

Methodology

Replicates

2 biological replicates per each ChIP experiment.

Sequencing depth

Please find the table ChIPseq_SequencingDepth.xlsx. in the source data file_Reporting summary sheet

Antibodies

- Anti-PHF5A (Proteintech, cat. 15554-1-AP; 1:200), https://www.ptglab.com/Products/PHF5A-Antibody-15554-1-AP.htm
- Anti-PHF14 (Proteintech, cat. 24787-1-AP; 1:200), https://www.ptglab.com/Products/PHF14-Antibody-24787-1-AP.htm
-Anti-KMT2A (Proteintech, cat. 29278-1-AP; 1:200), https://www.ptglab.com/products/MLL-Antibody-29278-1-AP.htm

Peak calling parameters

Peaks were called using MACS2 v2.2.7.1. Parameters: --format BAMPE --nomodel --shift -75 --extsize 150 -p 1e-3

Data quality

Raw reads were cleaned using fastp v0.23.2 with default parameters. Cleaned reads were confirmed high-quality using FastQC v0.11.9. Duplicated reads, reads mapped to ENCODE blacklisted regions , and reads with mapping quality lower than 30 were removed, and only properly paired reads were retained. The number of peaks are summarised in the table ChIPseq_batch1_peaks.xlsx and table ChIPseq_batch2_peaks.xlsx.

Software

BCL convert v4.0.3 was used to generate the fastq raw data. Raw reads were cleaned using fastp v0.23.2 with default parameters. Cleaned reads were then confirmed high-quality using FastQC v0.11. Burrows-Wheeler Aligner v0.7.17 was used to map cleaned reads to the human genome hg38. Duplicated reads, reads mapped to ENCODE blacklisted regions and reads with a mapping quality lower than 30 were removed, and only properly paired reads were retained. Peaks were then called using MACS2 v2.2.7.1. Common peaks within replicates were annotated with genes located within 5 kb upstream to 3 kb downstream of the gene body using ChIPpeakAnno v3.30.0. Differential binding analysis was performed using DiffBind v3.6.1, and peaks with adjusted p-value < 0.01 were considered significant.

Flow Cytometry

Plots

Confirm that:

☒ The axis labels state the marker and fluorochrome used (e.g. CD4-FITC).

☒ The axis scales are clearly visible. Include numbers along axes only for bottom left plot of group (a 'group' is an analysis of identical markers).

☒ All plots are contour plots with outliers or pseudocolor plots.

☒ A numerical value for number of cells or percentage (with statistics) is provided.

Methodology

Sample preparation

- Flow Cytometry analysis of enriched CSC surface markers in pancreatic tumorspheres: Single cells were suspended in ice-cold cell staining buffer consisting of DPBS (Thermo Fisher Scientific, cat. 14190169), 10% heat-inactivated FBS (Sigma-Aldrich, cat. F9665) and 0.1% sodium azide (Sigma-Aldrich, cat. S2002) then incubated with the Fc receptor blocking reagent human TrueStain FcX (Biolegend, cat. 422301) for 10 minutes at room temperature. Cells were labeled with fluorochrome-conjugated antibodies. Following labeling, cells were washed 3 times with the cell staining buffer then incubated with DAPI (BD Biosciences, cat. 564907) at a final concentration of 0.1 µg/ml in cell staining buffer for 15 minutes at room temperature in the dark for assessment of cell viability. Fluorescence minus one (FMO) and isotype control antibodies were used to gate and identify the positive cell population.

- Flow Cytometry analysis of SOX2 protein expression in PCSCs: We analyzed SOX2 protein expression levels by flow cytometry using the True-Nuclear transcription factor buffer set (BioLegend, 424401) and Pacific Blue anti-SOX2 antibody (BioLegend, 656111; 1:20) as per the manufacturer's instructions.

- Flow Cytometry analysis of apoptotic cells: Day 6 CSC-enriched pancreatic tumorspheres were harvested using a 40 µm cell strainer (pluriSelect, 43-50040-51) and dissociated with TrypLE Express enzyme (Thermo Fisher Scientific, 12604021). Single cells were seeded in ultra-low attachment 6 well plates at a density of 0.25 x 106 cells per well in stem cell-conditioned culture medium then treated with either DMSO (Sigma-Aldrich, D8418), MM-102 (Tocris Bioscience, 5307) at a final concentration of 50 or 75 µM, 10 µM OICR-9429 (Sigma-Aldrich, SML1209), or 10 µM MM-401 (MedChemExpress, HY-19554A) for 5 days in a humidified cell culture incubator at 37 °C and 5% CO2. To assess chromatin condensation as a

readout of apoptosis, cells were stained with Vybrant DyeCycle Violet and SYTOX AADvanced dyes (Thermo Fisher Scientific, A35135) as per the manufacturer's instructions. To distinguish between viable, early apoptotic, late apoptotic, and necrotic cells, pretreated PCSCs were stained with PE-Annexin V and 7-AAD (Biolegend, 640934) according to the manufacturer's instructions. Flow cytometry data were acquired using BD LSRFortessa cell analyzer (BD Biosciences), collected with FACSDiva software (BD, Version 8.0.1), and analyzed using FlowJo v10.8 software (BD Life Sciences).

Instrument

Flow cytometry data were acquired using BD LSRFortessa cell analyzer (BD Biosciences).

Software

Flow cytometry data were collected with FACSDiva Software (BD , Version 8.0.1) and analyzed using FlowJo v10.8 Software (BD Life Sciences).

Cell population abundance

Pancreatic cancer stem cells were sorted using magnetic activated cell sorting technique as follows: Pancreatic cancer cells were first suspended in ice-cold magnetic activated cell sorting buffer consisting of PBS, pH 7.2 (Thermo Fisher Scientific, cat. 10010023), 0.5% BSA (Sigma-Aldrich, cat. A7906), 2 mM EDTA (Thermo Fisher Scientific, cat. 15575020) and RevitaCell supplement (Thermo Fisher Scientific, cat. A2644501) then incubated with magnetic microbeads conjugated with anti-ABCG2 (Miltenyi Biotec, cat. 130-107-680; 1:5) and anti-SSEA4 (Miltenyi Biotec, cat. 130-097-855; 1:5) antibodies for 15 minutes at 4°C. Magnetically labelled cells were then sorted using an LS column (Miltenyi Biotec, cat. 130-042-401) inserted into a MediMACS separator (Miltenyi Biotec, cat. 130-042-302) as per the manufacturer's instructions. At passages 3-4, enrichment of pancreatic cancer stem cells in tumorspheres was tested by flow cytometry, where only batches showing at least 85% of CSC marker enrichment were used for subsequent experiments.

Gating strategy

FSC-A/SSC-A gates were used to eliminate cell debris from analysis > FSC-H/SSC-H gates were used to identify single cells > FSC-A/DAPI gates were used to eliminate dead (DAPI+) cells from the analysis. Both isotype and FMO controls were used to gate the positive cell population.

☒ Tick this box to confirm that a figure exemplifying the gating strategy is provided in the Supplementary Information.
